# Nutrition Literacy and Healthy Diet: Findings from the Validation of a Short Seniors-Oriented Screening Tool, the Spanish Myths-NL

**DOI:** 10.3390/ijerph182212107

**Published:** 2021-11-18

**Authors:** Elena Lobo, María Tamayo, Teresa Sanclemente

**Affiliations:** Faculty of Health Sciences and Sport, University of Zaragoza, Plaza Universidad, 3, 22002 Huesca, Spain; elobo@unizar.es (E.L.); mariata93@hotmail.com (M.T.)

**Keywords:** nutrition literacy, aged, questionnaires, Mediterranean diet, nutrition education

## Abstract

A good level of nutrition literacy (NL) is proposed as a determinant factor for following a healthy diet. Improving seniors’ NL might be particularly pertinent to enhance the quality of their diets. This study aimed to systematically design and validate a short seniors-oriented questionnaire as a screening tool to evaluate NL. We developed the Myths-NL questionnaire, composed of 10 widespread nutrition myths, and checked for its content and face validity. An observational cross-sectional study was conducted to explore the validity and the test–retest reliability, involving a community-dwelling group of 316 individuals aged 65 years and over. Construct validity was proved by establishing both discriminant and convergent validity. Cronbach α = 0.61 and Spearman r = 0.79 (*p* = 0.02) demonstrated internal consistency and test–retest reliability. Participants who had secondary/university studies scored significantly higher compared with those with primary (*p* < 0.001), and a significant linear relationship (R2 = 0.044, *p* = 0.001) with a positive slope (β = 0.209) between Mediterranean Diet Adherence Screener (MEDAS) and Myths-NL scores was observed, proving construct validity. In conclusion, the Myths-NL questionnaire is a valid and reliable tool to screen NL in Spanish seniors and it might be useful as an assessment NL tool for designing and implementing lifestyle interventions to promote healthy eating.

## 1. Introduction

It is well known that healthy eating behaviors depend on sociodemographic factors, general health status, and psychological/personal characteristics [1]. Nutrition literacy (NL) may be an overlooked contributor [2]. NL refers to the knowledge, skills, and competencies necessary to maintain nutritional health [3]. It focuses on the individual’s cognitive capacities, mainly basic literacy and numeracy skills, needed to understand and use nutritional information [4], especially considering that consumers receive this kind of data from multiple sources such as magazines, TV, and the Internet. It also involves the understanding of health risks and benefits of good nutrition, and the knowledge of the components of a healthy diet [5]. In fact, it has been shown that higher NL might predict better diet quality in adults with chronic disease [6].

It is known that elderly people have an increased rate of age-related diseases in which nutrition has an important role [7]. They are vulnerable to nutritional problems and deficiencies due to the physiological, psychological, and social changes in the aging process, affecting the ingestion, absorption, and metabolism of food, as well as the dietary choices [8,9]. Furthermore, restrictive or unbalanced diets are frequent in this group [10] and, many times, their culinary techniques are not adequate in either preserving the nutrient richness of food [11] or safeguarding food safety [12]. In fact, nutrition is considered a key element in the multidimensional concept of frailty. The nutritional frailty construct, referring to the condition commonly observed in vulnerable older adults that makes them susceptible to disability, has been recently operationalized as the presence of low body mass index (BMI) and skeletal muscle index (SMI), a higher dietary sodium intake, and lower dietary potassium and iron intakes [13].

This growing body of knowledge emphasizes the need to develop and implement effective lifestyle interventions, including diet quality improvement [7]. A review of behavioral interventions promoting healthy eating among older people showed that dietary educational interventions could lead to achieving a better dietary quality [14]. Recent research corroborates the fact that educational interventions, tailored towards enhancing NL, lead to improvements in dietary behaviors [15,16].

Therefore, focusing on seniors’ NL might be particularly pertinent, considering the challenge that the aged population represents worldwide, particularly in Western and Southern European countries in which this group and, more importantly, those called the oldest-old (80 or more years old), continues to grow [17]. However, there is little data on the level of NL in seniors, especially in the Mediterranean area, mainly because of the lack of appropriate instruments to measure it. On the one hand, there is a paucity of tools specifically designed for the elderly, and none of them is drawn up for Southern Europe [18]. On the other hand, although several questionnaires oriented to the general population have been recently adapted and validated in Mediterranean countries [2,19], those are difficult to administer to older people for whom simplicity and amenity of the format, easiness to understand, or quickness to complete are desirable aspects. Therefore, new, valid instruments for measuring NL targeting seniors living in Southern European countries would be useful as screening tools to help identify people at risk of low NL and to guide professionals on how to address dietary education.

Much of the information regarding food and nutrition that is presented as a fact in our neighborhoods is frequently composed of concepts poorly supported or contradicted by scientific evidence. Some of those misconceptions have been transmitted orally from generation to generation up to the present day, becoming myths [20]. Due to this fact, myths are supposed to be deeply rooted and established as beliefs in the elderly population, which could negatively influence their eating choices [21,22]. By definition, nutrition-literate individuals would be better skilled to handle information obtained from different sources, such as health professionals, family, friends, or diverse media [4], and they would be more critical concerning common misconceptions about food and nutrition. Thus, a questionnaire based on testing elderly beliefs about selected popular myths could be considered an easy way to assess the level of NL in this group. It would allow valuing the capacity to be critical with the information about nutrition both declarative, i.e., facts and procedures, and procedural, i.e., how to perform a task [5]. However, only a few of such tools are designed with this kind of item for Scottish [21], Swiss [23,24], American [25], and Slovak [26] populations, reflecting their specific dietary patterns and culinary traditions. Furthermore, these tools have been validated exclusively in adults.

Therefore, this study aimed to systematically design and validate a short, culturally sensitive, seniors-oriented questionnaire based on Spanish widespread nutrition myths as a screening tool to evaluate NL in a Southern European population.

## 2. Materials and Methods

### 2.1. Myths-Nutrition Literacy Questionnaire (Myths-NL): Items Generation

As the first step to develop the Myths-Nutrition Literacy questionnaire (Myths-NL), the authors and an external collaborator (E.F.) created a list of the 24 most repeated myths in the reviewed mass media sources of information about food, nutrition, and health (mainly Spanish bestseller magazines intended for adults, websites, and internet documents). Subsequently, 19 myths were chosen as the most influential ones on the elderly’s health, referring to nutritional value of food, intake frequency of food groups, hygienic matters, and culinary preparation. Five myths considered irrelevant for seniors were discarded (e.g., protein supplements to gain muscle). In an attempt to create a short, easy, and user-friendly instrument, the questions were formulated as true–false statements, and the items were formulated using the respondents’ usual language.

A group of experts composed of eight university lecturers from different areas and four health professionals reviewed the 19 items for content validity by commenting on both the statements and the questionnaire format. Based on their suggestions, that version was modified leading to a preliminary Myths-NL including 14 statements. Besides, to ensure face validity, the 14-item pool was piloted with seven elders (62–83 years old) to assess its readability and correct interpretation. The time of administration was also measured, and no more than 7 min were taken for its completion in any case. Only a vision problem was detected and, consequently, font size was increased.

### 2.2. Study Setting

The present study was conducted in Huesca (Aragón, Spain), a city with an elderly population of 10,243 inhabitants (19.7%). The convenience sample consisted of people fulfilling inclusion criteria: (1) aged 65 and over, (2) who were participating in municipal exercise activities for seniors, and (3) gave informed consent to participate voluntarily.

Two students of the Human Nutrition and Dietetics University Degree provided the anonymous questionnaire during one of the exercise sessions and, if necessary, assisted participants while they were answering. This questionnaire consisted of three parts:

- Part 1 included information about sociodemographic data (gender, age, education, and household type), self-reported level of physical activity, self-perceived health status, and the opinion about the importance of proper feeding and its impact on health. Additionally, participants were asked about which sources of food and nutrition information they consulted. They could choose between three possibilities and select as many options as they wanted: mass media, family and friends, and health professionals.
- Part 2 consisted of the previously cited preliminary Myths-NL. For each myth, participants had to decide if the statement was “true”, “false”, or “don’t know.” Based on the methodology described by some authors [21], correct answers scored one point, and incorrect, blank, or “don’t know” answers were scored as zero. Then, the total score was calculated, adding all item points.
- Part 3 incorporated the validated Mediterranean Diet Adherence Screener (MEDAS) developed within the PREDIMED study to assess the adherence to the Mediterranean Diet pattern. This questionnaire included 14 yes/no questions scored as 0 or 1. The final score ranged from 0 to 14. A total score of 9 or more points was indicative of adequate Mediterranean diet adherence [27].

### 2.3. Myths-Nutrition Literacy Questionnaire (Myths-NL): Internal Consistency, Reliability, and Validity

Firstly, as it is highly recommended [28], data was analyzed for item difficulty and item discrimination. Afterward, an evaluation of internal consistency of the Myths-NL final version was conducted.

To evaluate the test–retest reliability, one group of 15 participants answered the Myths-NL twice, with approximately a month difference between the first and the second administration.

Because of the lack of standards for measuring the aforementioned NL, construct validity was proved by establishing both discriminant and convergent validity [29]. To demonstrate discriminant validity, we assessed the Myths-NL’s capacity to distinguish populations assumed to have higher and lower levels of NL. Performance between groups with different education levels was compared, as respondents with primary studies were expected to have less critical capacity than those with secondary/superior studies. To assess convergent validity, we hypothesized that the Myths-NL score would be directly associated with dietary behavior, in terms of the adherence degree to the Mediterranean Diet pattern. For this latter analysis, participants living in nursing homes or hostels were excluded.

### 2.4. Statistical Analysis

Absolute frequencies and percentages, for categorical variables, and mean and standard deviation, for quantitative variables, were used in order to describe the characteristics of the sample. In an attempt to detect possible differences between the youngest-old and oldest-old, chi-squared and Fisher’s exact tests were calculated.

As far as questionnaire validation, frequencies of correct answers and Spearman item-to-total score correlations for each item were assessed to evaluate item difficulty (items that were answered correctly by less than 20% or by over 80%, i.e., too difficult or too easy) and item discrimination (item-to-total score correlation below ≈ 0.2, i.e., items with poor capability of discriminating between high- and low-scoring individuals), respectively. Internal consistency was assessed by Cronbach α; a value above 0.7 was considered adequate. Spearman and the intraclass correlation coefficients were used to compare the answers obtained in the study of repeatability, and values between 0.7–0.9 were considered as large correlations. To demonstrate discriminant validity, the score of the two groups of education level was compared by two-tailed t-test for independent samples and, also, multiple linear regression analysis was performed considering the sociodemographic variables significantly related to the Myths-NL score in the univariate analyses (i.e., age, education level, self-reported level of physical activity, and mass media as a source of information). Linear regression models were also performed to test the independent effect of Myths-NL score on MEDAS score, considering significantly correlated sociodemographic variables in the univariate analyses (i.e., gender, age, and household type).

Besides this, Spearman correlations between Myths-NL score and each MEDAS item were calculated.

All the analyses were performed using SPSS Statistics for Windows, Version 26.0 (IBM Corp., Armonk, NY, USA). Statistical significance was defined as *p* < 0.05.

## 3. Results

A total of 363 people attended the municipal sessions, from which 316 (87%) fulfilled the inclusion criteria and accepted taking part in the study. As shown in Table 1, participants were a community-dwelling group aged 74 (SD 6) years old, predominantly women, who reported an adequate level of physical activity. More than two-thirds had primary studies and good or excellent self-perceived health status. The great majority considered the usual diet as quite or very important to stay healthy. Compared with the youngest-old group (65–80 years old), the oldest-old had primary studies and lived alone or in a nursing home and had worse self-rated health more frequently. Regarding the sources of food and nutrition information, the most frequently marked option was health professionals (61%), followed by family and friends (45%) and mass media (35%). However, while the above was still true among the youngest-old group, family and friends became the most frequent source, followed by health professionals, among the oldest-old group.

Table 2 shows the 14 items included in the preliminary questionnaire, the frequency of correct answers, and the correspondent discrimination index. After applying the criteria for item difficulty and item discrimination, the final Myths-NL included 10 items and the total score had the Cronbach’s α of 0.61. As far as test–retest reliability, results for the overall score of the two attempts showed a value of Spearman’s correlation of 0.79 (*p* = 0.02) and the correspondent intraclass correlation coefficient of 0.66 (*p* = 0.02).

The Myths-NL score for the whole group showed a mean value of 5.8 (SD 2.0), with a range between 0 to 10. Statistically significant differences in the values obtained by youngest-old, 6.0 (SD 2.0), and oldest-old, 5.3 (SD 1.9), were found (*p* = 0.004).

Referring to discriminant validity, participants with a basic level of studies scored lower at the Myths-NL, 5.4 (SD 2.0), than those with secondary/university level, 6.9 (SD 1.9) (*p* < 0.001). Results of the multiple linear regression analyses showed that the level of education remained as a significant predictor of the Myths-NL score (β = 0.350; *p* < 0.001), as well as being oldest-old (β = −0.139; *p* < 0.001), physically active (β = 0.156; *p* = 0.004), and using mass media as one of the sources of nutrition information (β = 0.139; *p* = 0.01). The overall model fit was R2 = 0.197; *p* < 0.001.

As reported in Table 3, a significant linear relationship (R2 = 0.044, *p* = 0.001) between MEDAS and Myths-NL scores with a positive slope was observed (β = 0.209) as evidence of convergent validity. As indicated in Section 2.3, for this analysis, 21 participants who lived in residences or hostels were excluded. Those for whom the result of the MEDAS questionnaire was not available were considered missing data. No statistically significant differences were detected between the participants who were not included and those who formed the subsample (*n* = 244), except in the variable “living arrangement”. In this subsample, the MEDAS score showed a mean value of 9.1 (SD 2.0), which meant good adherence to the Mediterranean diet pattern.

According to the multiple linear regression analysis, higher estimated NL, living at home with others, being female, and being youngest-old were associated with higher adherence to the Mediterranean Diet pattern. It should be noted that the Myths-NL score showed the strongest independent association with the MEDAS score. Although in univariate analysis more variables were significantly related to MEDAS score, they did not remain significant in tested models. Thus, they were excluded from the final model (i.e., self-reported level of physical activity and mass media as a nutrition information source). The best-fitted model accounted for 13.6% of the variance in MEDAS score (*p* < 0.001).

Finally, the level of NL, measured as Myths-NL score, was significantly correlated with certain items of MEDAS (Table 4). Specifically, items related to vegetables and nuts, red meat or sausages, animal fat, sugar-sweetened beverages, and commercial pastries correlated positively with the Myths-NL score. On the other hand, it correlated negatively with the item related to legumes consumption.

## 4. Discussion

To the best of the authors’ knowledge, this is the first study that develops a short questionnaire based on widespread nutrition myths for estimating NL in seniors. It has shown to be a valid and reliable tool in a community-dwelling elderly population living in Huesca, a northeastern Spanish city. Myths-NL score was directly associated with diet quality (MEDAS score). Additionally, multivariate linear regression analysis confirmed that Myths-NL score, along with age, gender, and living arrangement, contributed to predict MEDAS score.

The Myths-NL was systematically designed and validated following the different steps proposed by Parmenter and Wardle [28]. Special attention was given to devising a short tool adapted to seniors. Firstly, little time was required to assess NL with this tool, avoiding this practical barrier [30]. Secondly, considering that the terminology may be a source of misunderstanding when communicating about health and food [31], common language was used along with the questionnaire. Finally, true/false queries led to a nonintimidating questionnaire that facilitated answering without pressure and allowed an objective measurement of the skills needed to understand and use nutrition information. In order to lessen the likelihood of random guessing, the questionnaire included the option “don’t know/don’t answer” [32].

The Myths-NL was developed from scratch, since other tools based on myths had been developed in countries with different food and culinary traditions [21,23,24,25,26]. Therefore, looking for misbeliefs in our cultural context implied a specific search strategy of Spanish food myths. Nevertheless, some of the items were very similar to the afore-referenced tools, such as those related to lentils (n° 1), fat (n° 3), or oily fish (n° 10), suggesting its widespread importance.

The final Myths-NL consisted of 10 items after the exclusion of four initial elements without reducing content validity. Myths related to handwashing (n° 13), oily fish (n° 10), and pastry products (n° 9) were removed because over 80% of respondents answered them correctly and showed poor item discrimination. Customs and traditions, such as adequate personal hygiene or preserving familiar recipes, constitute forms of social expression of seniors’ life. Besides, it seems they have a sound knowledge of the health benefits of oily fish [33]. Additionally, the item related to the daily intake of eggs (n° 8) with 31% of correct answers was discarded because its discrimination index value was near 0.2. In this sense, this could be reflecting that egg consumption has been traditionally widely discouraged by health professionals even when there is still no broad consensus about its effects on health [34]. It is worth noting that the item referring to canned food (n° 14), with 11% of correct answers and discrimination index value nearer to 0.3, was kept based on its content about food safety [12]. Finally, myths related to dairy with several correct answers slightly higher than 80% were maintained due to their adequate discrimination index and their importance in the health of the elderly [11,12,35].

The internal consistency of Myths-NL was acceptable and allowed to build an overall sum score. The Cronbach α value, slightly lower than 0.7, is consistent with results in the literature for brief instruments with narrow scale width [25,26]. Besides, it is known that scales designed to measure constructs that imply a wide range of topics must have some degree of heterogeneity among the items and, consequently, lower values for Cronbach α [36]. Possible future revisions of the questionnaire should consider the improvement of this psychometric property.

Correlations revealed good test–retest reliability [28], indicating that the results were consistent over time. Although higher test values might be expected [25,30], the age of our sample and the more expanded time interval between both administrations, compared to the 2 weeks often used [28], could explain our results.

When discriminant validity was tested, the results showed that those individuals with lower levels of education have significantly less NL than more educated participants. Other authors have shown similar associations, and of comparable magnitude, between lower educational attainment and poor health and NL [24,30], even in the elderly population [37,38]. Moreover, the multivariate regression model underpins these results, as those with secondary/university education level still score higher after controlling for age, self-reported physical activity, and the use of mass media as one of the sources of food information. Precisely, it is interesting that, in this study, mass media use was associated with a higher Myths-NL score. This is congruent with the positive association between the use of media channels (TV, newspapers or magazines, and internet) and the NL level shown in other studies where nutrition education interventions were carried out [3], probably referring to an active and determined effort to obtain nutritional information [39].

As evidence of convergent validity, the Myths-NL score was the strongest predictor of Mediterranean diet adherence, as previously shown for other NL measurement tools in the literature [2,6,25,40]. The low values in statistical parameters of the regression analyses and the correlation coefficient between Myths-NL and MEDAS tools have been observed previously, suggesting that the association between NL and dietary behavior is weak [6,24]. In terms of association, small associations, which are consistently and widely observed, are still valid [41].

Our results also showed that the Myths-NL score correlated with affirmative responses about taking adequate amounts of several food categories, such as vegetables, red meat or sausages, animal fats, sugar-sweetened beverages, commercial pastries, or nuts. This could be attributed, in part, to nutrition education and communication efforts in which recommendations of intake may have been successfully understood by those more literate. In spite of that, the selection of foods that make up an individual’s diet is influenced by a large number of factors. In addition, these factors can have more or less relevance depending on the food group that is assessed [42]. The negative correlation with legume intake was unusual because, although the results showed that its nutritional value was known, other factors—the appearance of flatulence or abdominal discomfort, the high cooking time required, or the perception of beans being “a poor man’s meat”—may discourage its consumption [43].

The present study has several limitations. The sample was mainly composed of physically active older women in good self-reported health. This fact should not greatly affect the validation process, given the consistency of the data concerning other studies, and could therefore be appropriate for other older population groups in Spain, even in other Mediterranean countries. Nevertheless, robust construct validity requires evidence from multiple studies, necessitating that this tool would further be tested in similar samples and in populations that deviate from this sample. Both NL and adherence to the Mediterranean diet were measured successively in the same questionnaire. Consequently, the latter might be reflecting the participant’s ideas rather than their actual behavior. This is a common limitation in the field of behavioral nutrition research, which uses surveys that measure behavioral determinants and dietary behavior [44].

## 5. Conclusions

The Myths-NL questionnaire designed and tested in the current study is a valid and reliable screening tool to evaluate NL in Spanish seniors. Due to the cultural proximity to other southern European countries, its use could also be appropriate in other elderly populations in the Mediterranean area. Furthermore, our results support the idea that NL is associated with diet quality. Therefore, the Myths-NL developed in this study might be useful as an assessment NL tool for designing and implementing lifestyle interventions to promote healthy eating.

## Figures and Tables

**Table 1:** Self-reported characteristics of study sample in the validation of the Myths-Nutrition Literacy Questionnaire, Myths-NL.

	All		Youngest-Old		Oldest-Old		
	*n*	%	*n*	%	*n*	%	*p*
Gender							0.624
Male	29	9	21	9	8	11	
Female	285	91	218	91	67	89	
Education level							0.032 ^1^
Primary	210	68	153	65	57	78	
Secondary/University	100	32	84	35	16	22	
Living arrangement							<0.001
At home alone	100	33	65	28	35	48	
At home with others	186	61	157	67	29	40	
Nursing home/Hostel	21	7	12	5	9	12	
Physical activity							0.097
<3 times/week	74	23	51	21	23	31	
≥3 times/week	240	76	188	79	52	69	
Self-rated health							0.009 ^1^
Excellent or good	217	69	175	73	42	57	
Moderate or poor	96	31	64	27	32	43	
Importance diet for health							0.450
Not much/A little	37	12	30	13	7	10	
Quite/Very	273	88	206	87	67	90	
Mass media ^2^	104	35	81	36	23	31	0.499
Family and friends ^2^	134	45	90	40	44	60	0.003 ^1^
Health professionals ^2^	181	61	149	66	32	44	0.001 ^1^

Differences between youngest-old (65 and 80 y old) and oldest-old (equal to or more than 80 y old) tested by chi squared or ^1^ Fisher’s exact test, when appropriate. ^2^ Frequency this source of information was selected.

**Table 2:** List of the preliminary Myths-NL questionnaire with their correct answer, the percentage of respondents answering correctly and the correspondent discrimination index (*n* = 316).

No. Item	Myths	True/False	% Correct Answer	Discrimination Index
1	Lentils have a high iron content but nothing else	F	37	0.60
2	Whole grain bread is less fattening than white bread	F	47	0.51
3	Fat is always bad for health, whatever the type	F	36	0.50
4	When chocolate is “sugar free” we can eat all we want	F	79	0.52
5	After binging, fast for a day	F	65	0.53
6	Adults do not need to drink milk nor eat other dairy products such as yoghurt or white cheese	F	81	0.46
7	Fish nourishes the same as meat	T	77	0.32
8	**Eating eggs every day is banned because they are very high in cholesterol**	F	31	0.22
9	**If we eat pastry products, they should be homemade**	T	87	0.10
10	**Salmon and sardines are very healthy for the heart**	T	96	0.15
11	Nuts have many calories so that it is not recommended to eat them	F	69	0.47
12	The way in which food is prepared and seasoned influences the amount of nutrients in the final meal	T	83	0.29
13	**It is important to wash your hands with soap before and after eating**	T	99	−0.02
14	Home-made canned food is always better than commercial	F	11	0.29

Discrimination index calculated by rho Spearman. In bold letters, myths rejected after applying the exclusion criteria.

**Table 3:** Regression analysis between estimated nutrition literacy, sociodemographic factors and adherence to Mediterranean diet pattern (*n* = 244).

	Adherence Degree Mediterranean Diet Pattern				
	Unstandardized β			Standardized β	*p*
	β	Dev. Error	95% CI		
MODEL 1					
Constant	7.793	0.400	7.005–8.580	-	<0.001
Myths-NL	0.208	0.063	0.085–0.331	0.209	0.001
MODEL 2					
Constant	6.139	0.572	5.013–7.266	-	<0.001
Myths-NL	0.184	0.061	0.065–0.303	0.185	0.003
Gender					
Male	Reference				
Female	1.062	0.413	0.247–1.876	0.156	0.011
Age					
Oldest-old	Reference				
Youngest-old	0.859	0.297	0.275–1.443	0.180	0.004
Living arrangement					
At home alone	Reference				
At home with others	0.617	0.258	0.108–1.126	0.148	0.018

**Table 4:** Affirmative answers to each of the MEDAS questions and correlations with the Myths-NL score (*n* = 244).

	Affirmative Answers		Correlations with Myths-NL Score	
	*n*	%	r	*p*
1. Using olive oil as the principal source of fat for cooking	239	98	0.058	0.368
2. ≥4 tablespoon of olive oil/day (e.g., used in frying, salads, meals eaten away from home)	137	56	0.009	0.892
3. 2 or more servings of vegetables/day (one serving = 200 g)	183	75	0.199	0.002
4. 3 or more pieces of fruit/day	189	78	−0.069	0.280
5. ≤1 serving of red meat or sausages/day (one serving = 100–150 g)	175	72	0.182	0.004
6. ≤1 serving of butter, margarine or cream/day (one serving = 12 g)	188	77	0.144	0.024
7. <1 of sugar-sweetened beverage/day	194	80	0.190	0.003
8. ≥7 servings of red wine/week	33	14	0.107	0.097
9. ≥3 servings of legumes/week (one serving = 150 g)	66	27	−0.164	0.010
10. ≥ 3 servings of fish or seafood/week (one serving of fish = 100–150 g and seafood = 200 g)	147	60	0.074	0.249
11. <2 commercial pastries/week	133	55	0.160	0.012
12. 3 or more servings of nuts/week (one serving = 30 g)	132	54	0.190	0.003
13. Consuming white meat (e.g., poultry) over red meat (e.g., cow, pig)	216	89	−0.049	0.444
14. ≥2 servings/week of a dish with a traditional sauce made of tomatoes, garlic, onion, or leeks sauteéed in olive oil (*sofrito*)	178	73	−0.091	0.155

## Data Availability

The data presented in this study are available upon reasonable request to the corresponding author.
